# Varying Synthesis Parameters of Potato Starch Aerogel for Aerospace Applications

**DOI:** 10.3390/gels11060467

**Published:** 2025-06-18

**Authors:** Jacob Staker, Daniel A. Scheiman, Janice Mather, Jamesa L. Stokes, Haiquan Guo

**Affiliations:** 1 Department of Chemistry, Purdue University, West Lafayette, IN 47907, USA; 2 Universities Space Research Association, Cleveland, OH 44135, USA; 3 University of Akron, Akron, OH 44325, USA; 4 NASA Glenn Research Center, Cleveland, OH 44135, USA

**Keywords:** potato starch, aerogel, biodegradable, thermal insulation, sustainability

## Abstract

Aerogels have the potential for usage in many daily and high-tech aerospace applications. Silica aerogels are fragile, while organic aerogels are much tougher, but they are both generally synthesized using toxic solvents. Biodegradable aerogels, if they possess similar properties as polymer aerogels, could be widely utilized in many aerospace applications and offer environmental benefits. In this work, potato starch aerogels were systematically studied. The potato starch concentration, the amount of plasticizer (glycerol), and an acid source (acetic acid) were varied. The relationship of the precursors on potato starch aerogel’s properties, such as density, shrinkage, porosity, BET surface area, mechanical properties, and thermal conductivities, were researched. The resulting potato starch aerogels possess suitable density, Young’s modulus, and thermal conductivity for use in many aerospace applications.

## 1. Introduction

Aerogels are highly sought-after energy-efficient materials that can be used for many applications, especially as thermal insulation material for aerospace due to their lightweight and low thermal conductivity [1]. Silica aerogels and silica-based aerogels in particular are commonly used in this field, as they are capable of retaining their mesoporous structure at high temperatures. However, they are mechanically brittle and fragile, which limits their feasibility for continuous use [2]. Many other aerogels have also been developed, including carbon aerogels [3], polyimide aerogels [4,5,6,7,8,9,10], polyamide aerogels [5], and polyurethane aerogels [6]. Since 2009, polyimide aerogels have opened up more opportunities for various aerospace applications, such as thermal insulation [7], nanogenerators [8], antenna substrates [9], and IR scattering filters [10]. However, to fabricate polyimide aerogels, toxic organic solvents like n-methylpyrrolidone (NMP), dimethylformamide (DMF), and dimethylacetamide (DMAc) are generally used [11]. The United States Environmental Protection Agency (EPA)’s risk evaluation in 2020 found that NMP can cause serious health effects, such as miscarriages and reduced fertility. Furthermore, NMP can cause harm to many organs and bodily systems, like the liver, kidneys, immune system, and the nervous system. Because of these risks, in June 2024, the EPA announced a proposed rule under the Toxic Substances Control Act (TSCA) and an NMP Workplace Chemical Protection Program (WCPP). If finalized, this will restrict the usage of NMP in the near future due to concerns over workers’ health [12]. In addition to the syntheses involving organic toxic solvents, many polymers are difficult to decompose, causing environmental harm [13]. Many organizations, like the EPA, across the globe are fighting to protect the environment. Environmentally friendly processes are currently on the rise in many commercial fields, including the aerospace sector, which desire sustainable materials to reduce environmental impact and enhance the recyclability of components.

Making substitute aerogels using non-toxic precursors and solvents is a possible way to mitigate the aforementioned issues. The synthesis of bio-based aerogels generally involves using non-toxic solvents, such as water and ethanol. The lack of toxic solvents and the main composition of natural biopolymers make these materials a promising sustainable alternative to standard aerogel chemistries for aerospace applications. Starch, as one of the most abundant biomaterials, has been fabricated into aerogels and explored extensively [14,15,16,17,18]. Currently, most research has been focused on the retrogradation process [15], the effect of amylose and amylopectin on the final properties of aerogels [16], and fabrication processes with different drying methods [17]. Starch aerogels have also been investigated for potential applications in thermal insulation, drug delivery, and packing materials [14,18,19]. In particular, potato starch has been the most studied among all starch aerogels.

While potato starch aerogels have been widely studied, no systematic study of the synthesis parameters has been conducted. Furthermore, the evaluation of their potential usage in aerospace applications has not been investigated either. The goal of this work is to evaluate and optimize the properties of potato starch aerogels such as density, thermal conductivity, thermal stability, and mechanical properties required for potential aerospace applications. For this purpose, a design of experiments was used to study the relationship of synthesis parameters (e.g., potato starch concentration, acetic acid amount, and glycerol amount) with the properties of potato starch aerogels (density, surface area, thermal conductivity, and compressive modulus). Potato starch gels were fabricated by reacting a mixture of acetic acid, glycerol, and potato starch in water at 100 °C, followed by room temperature aging, −20 °C freezing, and ethanol washing. Potato starch aerogels were then produced by the supercritical extraction of the wet gels with CO_2_. The resulting potato aerogels had densities of 0.11–0.30 g/cm^3^, compressive moduli of 2.7–37.8 MPa, and thermal conductivities of 37.5–45.2 mW/mK, which were comparable to reported polyimide aerogel properties [4,5,6,7,8,9,10].

## 2. Results and Discussion

**Figure 1:**
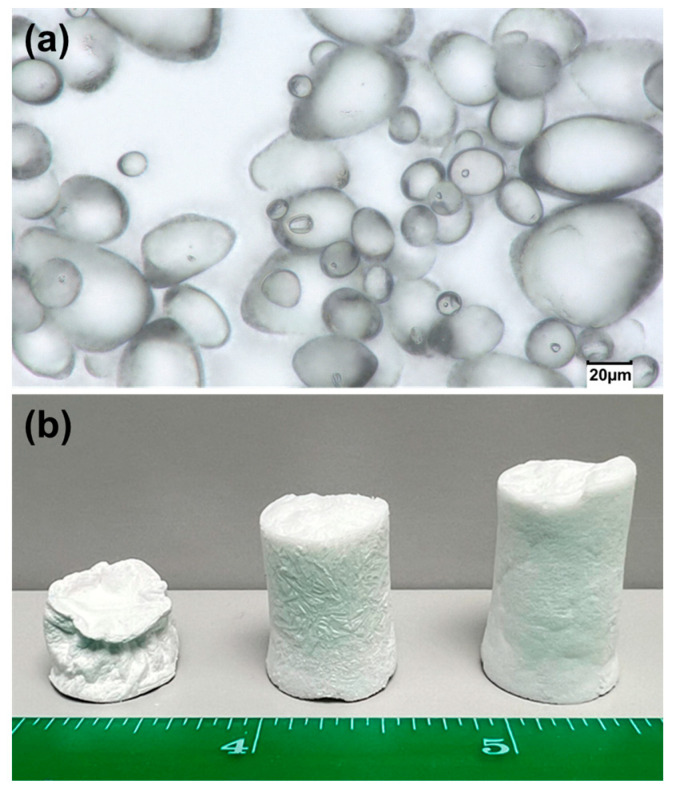
(**a**) Optical microscope image of pure potato starch and (**b**) potato starch aerogels that were prepared by (left) being directly soaked in ethanol after one day of RT aging; (middle) one day frozen at −20 °C directly without one day of RT aging, and then followed with ethanol washing; (right) one day of RT aging, one day frozen at −20 °C, followed by ethanol washing.

Figure 1a shows an exemplary optical image of the potato starch precursor used in this study. As seen in the image, the potato starch precursor exhibited a mix of oval-, irregular, and round-shaped granules ranging from 5 to 55 µm in size. For the initial study, the potato starch aerogels were prepared by heating the mixture of potato starch, acetic acid, glycerol, and water at two different temperatures as follows: 60 °C and 100 °C. Gels made from mixtures at 100 °C were sturdier than those made at 60 °C and had higher surface areas. The retrogradation and solvent processes were further studied for the gels made at 100 °C. As seen in Figure 1b, when the gel was washed directly in ethanol after one day of aging at room temperature (RT), the surface/skin of the aerogel became wrinkled and deformed. When the gel was directly put into the freezer (−20 °C), followed by ethanol washing without one day of RT aging, icicles formed on the surface of the aerogel. Finally, the sample that was aged for one day, frozen for one day (at −20 °C), and then washed five times with ethanol had the most uniform structure with no icicle formation.

Retrogradation during the sol–gel process is normal for starch, where a more ordered structure is formed due to the relaxation of the biopolymers. The fast retrogradation rate happens at 0–4 °C [20]. From the observed morphology of the synthesized gels, RT aging and freezing were both necessary for the retrogradation process to be completed. If the gel was directly soaked in ethanol, the retrogradation process was disrupted, resulting in a wrinkled and distorted sample shape. Also, if the sample was directly frozen without RT aging, icicles formed at the surface of the aerogel, as the ice-forming rate was faster than the retrogradation rate. From the authors’ previous study of preparing mixtures at 60 °C, it was observed that freezing the sample for two days significantly reduced the aerogel shrinkage [21] due to the completion of the retrogradation process accompanied by ice growth, which expanded the gel structure. The expansion of the structure from the ice formation happened during the second day of deep freezing. For this study, it was important to investigate the effect of synthesis parameters on the properties of the aerogels while ignoring the ice-expanding effect during the second day. Thus, all the samples in this paper were prepared at 100 °C, aged at RT for one day, frozen at −20 °C for one day, and then washed with ethanol five times.

The potato starch aerogels were prepared by varying the concentration of potato starch from 5–10 wt%, the potato starch to acetic acid ratios (g/g) from 25 to 75, and the glycerol to potato starch ratios (g/g) from 0.5 to 0.75. The detailed formulations and the properties of the resulting potato starch aerogels are listed in Table 1.

**Figure 2:**
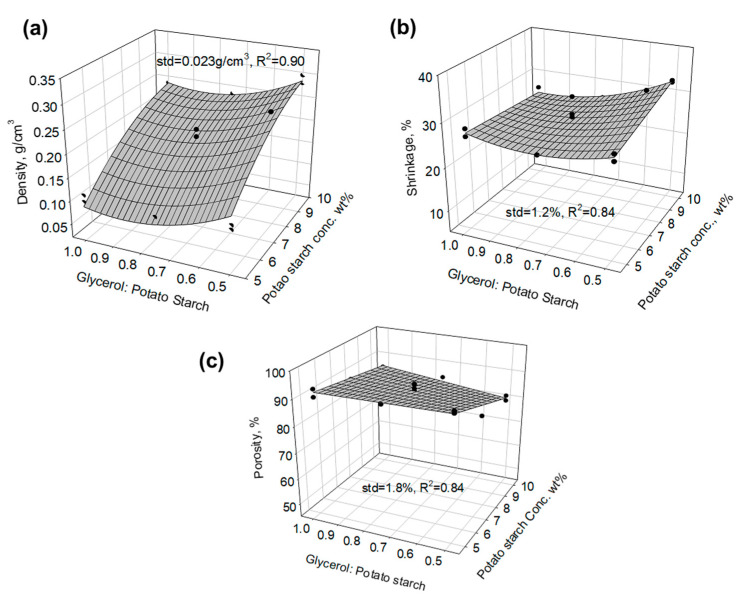
Empirical model plots of the relationship of (**a**) density, (**b**) shrinkage, and (**c**) porosity showing that glycerol amounts and potato starch concentration are impact factors.

The densities of the potato starch aerogels ranged from 0.104 g/cm^3^ to 0.295 g/cm^3^, the shrinkages ranged from 24.6% to 36%, and the porosities ranged from 77.6% to 92.8%. Figure 2 shows empirical models for the relationships of density (standard deviation = 0.023 g/cm^3^, R^2^ = 0.90), shrinkage (standard deviation = 1.2%, R^2^ = 0.84), and porosity (standard deviation = 1.8%, R^2^ = 0.84) with potato starch concentration, potato starch to acetic acid ratio, and glycerol to potato starch ratio. Both the potato starch concentration and glycerol amount effected the density, shrinkage, and porosity of the aerogels. Acetic acid, which acts as a chain breakage agent, has no effect on density, shrinkage, and porosity. With an increase in potato starch concentration, the density increased significantly, the corresponding porosity decreased, and the shrinkage increased slightly at lower glycerol amounts but decreased at higher glycerol amounts. A 0.8 glycerol-to-potato starch weight ratio at 5 wt.% potato starch resulted in the lowest density aerogel. As the glycerol amount increased, the shrinkage of the aerogels decreased. At the lower potato starch concentration, the glycerol amount did not have a significant effect on the porosity. However, at higher potato starch concentrations, as the glycerol amount increased, the porosity increased. The amount of potato starch determined the mass of the aerogel monolith. Glycerol crosslinks the starch chains, which affects the final volume of the aerogel. The combination of these two, potato starch concentration and glycerol amount, affected the final density, shrinkage, and porosity, which in turn influenced the observed trends.

**Figure 3:**
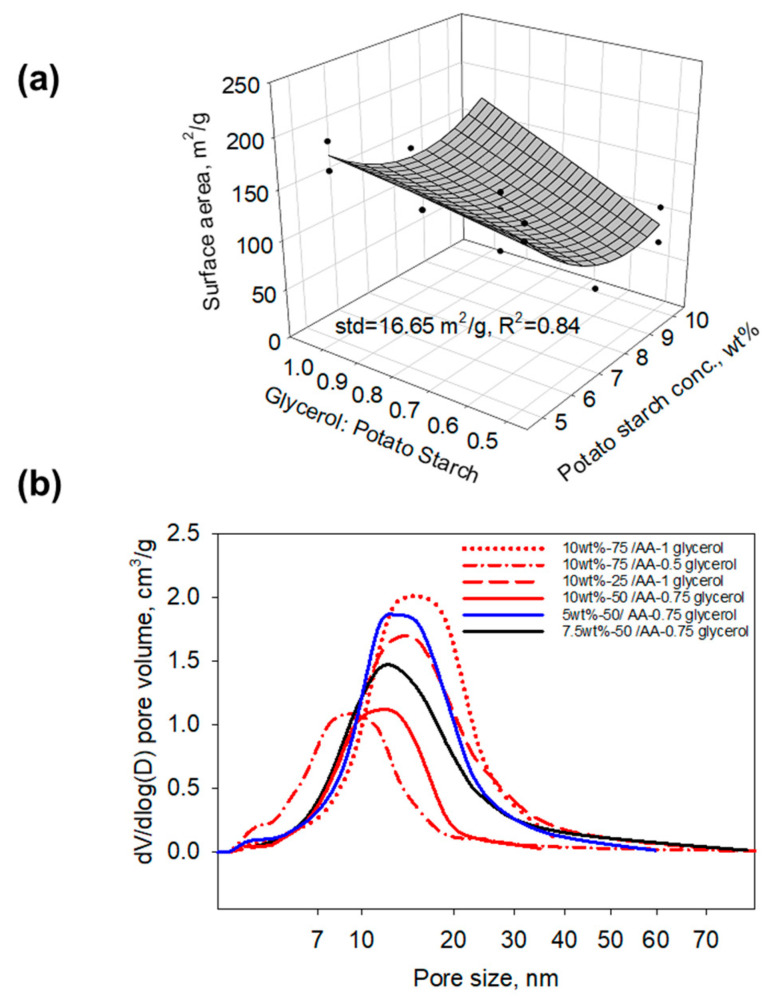
(**a**) Empirical surface area of the potato starch aerogels indicating that the surface area depends on the amounts of glycerol and potato starch concentration; (**b**) pore size and pore volume distribution of the potato starch aerogels.

N_2_ adsorption/desorption was conducted to study the surface area and pore size distribution of the samples. Figure 3a shows the empirical model plots of the Brunauer–Emmett–Teller (BET) surface area of the aerogels (standard deviation = 16.65 m^2^/g, R^2^ = 0.84). The surface areas of the samples ranged from 70.3 to 198.6 m^2^/g. As the glycerol amount increased, the surface area increased. The potato starch concentration also had an effect on the surface area. A concentration of 7.5 wt% potato starch resulted in the lowest surface area. Figure 3b shows the pore size–pore volume distribution plot of the potato starch aerogels. The pore volume and major pore size increased as the potato starch concentration decreased, which is similar to the porosity trend observed in Figure 2c. With acetic acid increasing, pore volume decreased. Increasing glycerol amounts resulted in an increase in pore volume and a larger major pore size. For example, the formulation with 10 wt% potato starch, a potato starch/acetic acid weight ratio of 75, and a glycerol/potato starch weight ratio of 0.5 had a major pore size of 8.7 nm and a BJH desorption cumulative pore volume of 0.46 cm^3^/g. Meanwhile, when adjusting the glycerol/potato starch weight ratio to 1 and keeping the other two parameters the same, the aerogel had a major pore size of 15.6 nm and a BJH desorption cumulative pore volume of 0.84 cm^3^/g.

**Figure 4:**
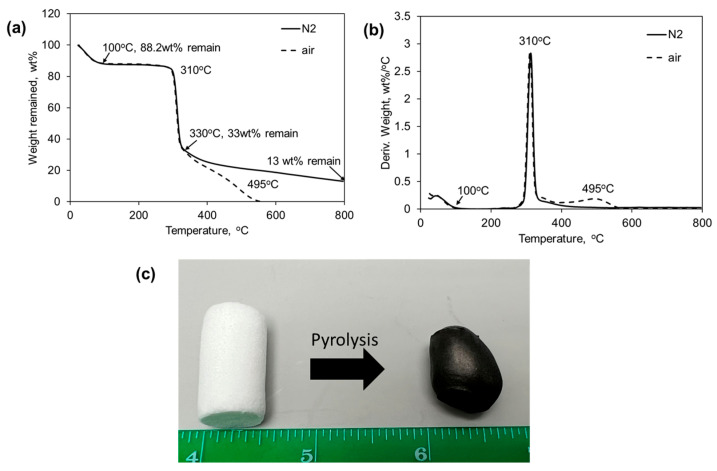
(**a**) Typical TGA curves of potato starch aerogels analyzed in N_2_ and air; (**b**) typical derivative thermogravimetric curve of potato starch aerogels in N_2_ and air; and (**c**) photo of a carbon aerogel (right) pyrolyzed from the potato starch formulation of 10 wt% potato starch, potato starch/acetic acid = 50 g/g, and glycerol/potato starch = 0.75 g/g.

**Figure 5:**
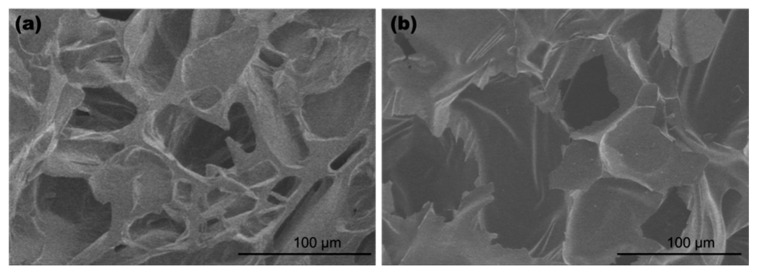
Scanning electron microscope images of (**a**) potato starch aerogel with 10 wt% potato starch, potato starch/acetic acid = 50 g/g, and glycerol/potato starch = 0.75 g/g; (**b**) carbon aerogel pyrolyzed from aerogel (**a**).

Figure 4a shows the typical thermogravimetric analysis (TGA) curves of the potato starch aerogels analyzed in nitrogen and in air. There was an initial solvent and moisture loss before 100 °C. In Figure 4b, showing the derivative thermogravimetric curves at 310 °C, the potato aerogels exhibit immense mass loss, indicating decomposition occurs at this temperature. In air, there was a second decomposition step observed at 495 °C, which was probably the onset of oxidation of the remaining char after 310 °C. The potato starch aerogels had a char yield of about 13–20 wt% in N_2_ at 800 °C. Although lower than the polyimide aerogel’s char yield (>55%) in N_2_ [4], carbon aerogels could still be yielded by heating the potato starch aerogel at 800 °C in argon gas, as seen in Figure 4b. Carbon aerogels can have a higher application temperature up to 2500 °C in inert gas or vacuum environments [22], so they can be potentially used in aerospace and energy storage applications where high temperature stability is required. Using potato aerogels to produce carbon aerogels is a more sustainable method than the pyrolysis of other polymer aerogels. The carbon aerogel did not have a uniform diameter but contained a stable structure. It was observed that the carbon aerogel shape could be controlled by the crucible shape used and heating temperature, along with the heating and cooling rate, which might affect the outgassing during the pyrolysis process. As seen in the scanning electron microscope (SEM) images (Figure 5), the pore sizes of carbon aerogels in the macropore ranges increase due to the release of CO_2_, CO, and H_2_O during the pyrolysis process. The surface area of the carbon aerogel decreased from 156.1 m^2^/g to 86.9 m^2^/g, which was due to the decrease in the pore volumes of 0–100 nm during the pyrolysis.

**Figure 6:**
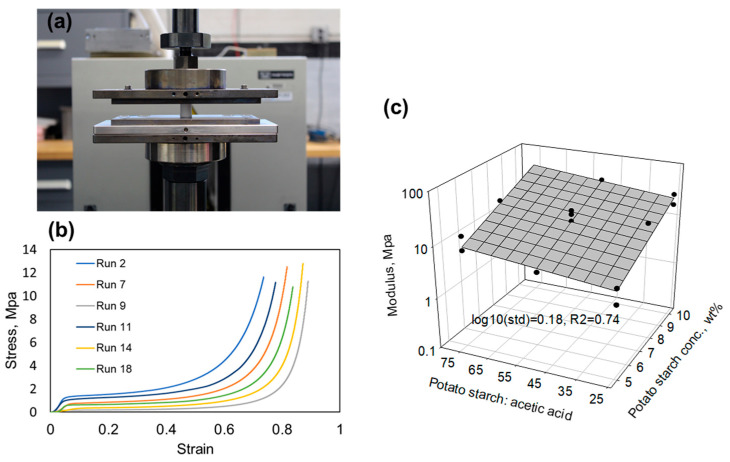
(**a**) Image of a potato starch aerogel in the compression test setup; (**b**) the stress–strain curves of potato starch aerogels; (**c**) empirical model plot of the compression Young’s moduli of potato aerogels exhibiting that Young’s moduli are affected by the acetic acid amount and potato starch concentration.

Potato starch aerogel cylindrical monoliths were prepared for compression testing, as shown in Figure 6a. At a constant displacement rate of 0.05 in./min, the aerogel was compressed until a measured force of 450 lbf. Figure 6b shows the selected stress–strain curves from the compression test of the potato starch aerogels. The Young’s modulus was calculated from the initial slope of the elastic region of the aerogels. The Young’s moduli of the aerogels ranged from 2.7 MPa to 37.8 MPa, which was higher than that of silica aerogels and comparable with some polyimide or polyamide aerogels [5,23]. For instance, at a similar density, 1,3,5-triaminophenoxybenzene (TAB) crosslinked polyimide aerogels made with benzophenone-3,3′,4′4′-tetracarboxylic dianhydride (BTDA) and 4,4′-oxydianiline (ODA) have Young’s moduli around 0.9–2.3 MPa, and TAB crosslinked polyimide aerogels made with p-phenylene diamine (PPDA) and biphenyl-3,3′,4,4′-tetracarboxylic dianhydride (BPDA) have Young’s moduli 19.1–46.1 MPa. As a result, potato starch aerogels may potentially have wider applications than silica aerogels and are more sustainable than polyimide aerogels. As seen from the empirical model plot (Figure 6c) of the Young’s moduli (log10 (standard deviation) = 0.18, R^2^ = 0.74) of the potato starch aerogels, a higher potato starch concentration resulted in a higher elastic modulus. Unlike density, the glycerol amount had no significant effect on the modulus. Instead, the acetic acid influenced the modulus, where increasing acetic acid resulted in a slight modulus decrease. Acetic acid helps reduce the branch structure of amylopectin in the potato starch and shifts to a more linear amylose-like structure, causing a decrease in the modulus. Although glycerol crosslinks the potato starch chains, due to its much shorter linkage compared to potato starch, the strengthening effect could not be observed by increasing the glycerol amount.

Because the potato starch aerogels have a large amount of hydroxyl groups on the surface of the aerogels, water can be absorbed very quickly. However, an aerogel that floated on water was observed to remain floating after one week. At this time, the aerogel was removed from the water so that the total time the aerogel could float was speculated to be longer. The structure of the aerogel remained intact with a little swell in size. Water was trapped inside the pores. In addition, no significant structural change was observed when it was exposed to moisture in the air after a year. Since the structure of potato starch aerogel can be retained in water, a simple solvent exchange in ethanol can remove the water and return to an aerogel again. For applications where the aerogel needs to be hydrophobic, the backbone of the potato starch aerogel can be modified with aliphatic groups [24]. Also, hydrophobicity can help prevent possible adverse behavior, like mold growth caused by moisture retention. Future research can include the modification of potato starch aerogels with aliphatic groups.

**Figure 7:**
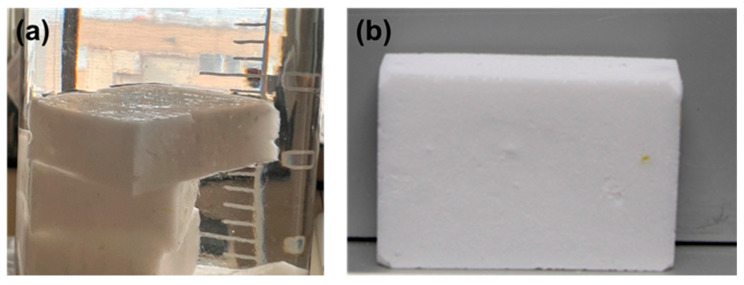
(**a**) Wet gels made from recycled potato starch aerogel powders; (**b**) potato aerogels made from the wet gels in (**a**) to prove recyclability.

In aerospace applications, long-life durability is necessary. Potato starch is an abundant resource on earth and a biodegradable material. Starch should be easy to decompose [25] into glucose, but as previously described, potato aerogels did not show structure change after exposure to air moisture for one year. For this research, it is impossible to study life duration. It is expected that potato starch aerogel will be used as inside layers in potential applications; thus, degradation caused by moisture and UV irradiation can be reduced. However, it was interesting to observe that potato starch aerogels can also be recycled. The potato starch aerogels can be grounded into powders that can be reused as the precursor. Following the same sol–gel process, the powder was reformed into new potato starch aerogels. Figure 7 shows the wet gels made from the recycled potato aerogel powders and the resulting new aerogels. The recyclability of potato starch aerogels could also help reduce waste accumulation in the environment, making these materials even more sustainable for various applications.

**Figure 8:**
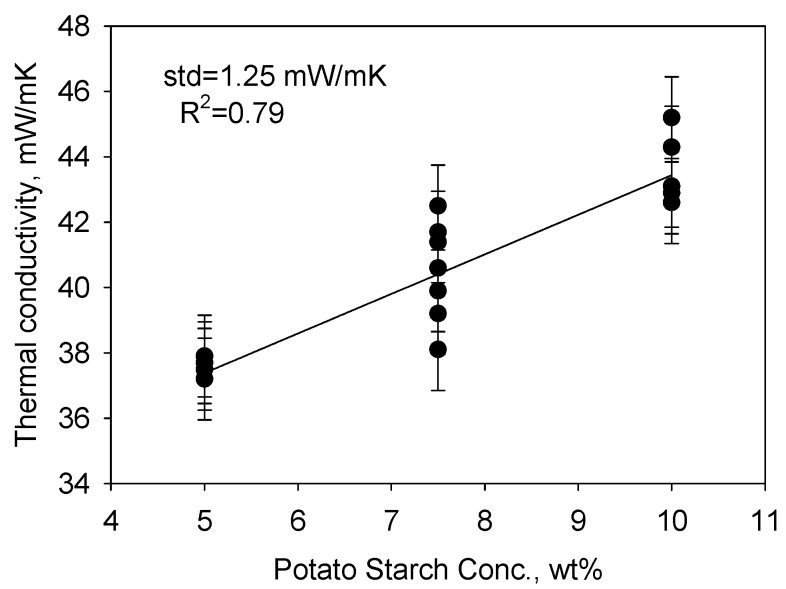
Empirical model plot of the thermal conductivity of potato starch aerogels indicating that only potato starch concentration affects thermal conductivity.

The measured thermal conductivities of the potato starch aerogels ranged from 37.5 mW/mK to 45.2 mW/mK. The empirical model plot of the thermal conductivity (standard deviation = 1.25 mW/mK, R^2^ = 0.79) of the potato starch aerogels is shown in Figure 8. The thermal conductivity greatly depended on the potato starch concentration, but not on the amounts of acetic acid or glycerol. Higher potato starch concentrations resulted in higher thermal conductivities. The thermal conductivity of the potato starch aerogels was also higher than silica aerogels (12–20 mW/mK) [26] but was close to some reported polyimide aerogels (for example, 30.8 mW/mK, reported by Feng, J. et al. [27], and 39.89 mW/mK, reported by Yang, J. et al. [28]) and lower than many insulation materials [29] currently in use.

## 3. Conclusions

Potato starch aerogels were made with 5–10 wt% potato starch; potato starch/acetic acid ratios ranged from 25 to 75 g/g, and glycerol/potato starch ratios varied from 0.5 to 1 g/g. The aerogels made by utilizing the higher-temperature process were sturdier and had higher surface areas. It was found that both room-temperature aging and deep-frozen processes were necessary to make well-structured aerogels. Potato starch concentration has a great impact on all the properties investigated. Acetic acid had no effect on density, shrinkage, porosity, or surface area, but affected pore volumes and mechanical properties. The glycerol amount slightly affected density, shrinkage, porosity, pore sizes, and surface area, but not Young’s modulus. Thermal conductivity only depended on potato starch concentration. The resulting potato starch aerogels were not only biodegradable, recyclable, and economical, but also their density (0.11–0.30 g/cm^3^), mechanical modulus (2.7–37.8 MPa), and thermal conductivity (37.5–45.2 mW/mK) were all applicable for thermal insulation materials, comparable to polyimide aerogels. Although the potato starch aerogels absorb water easily, they retain their structure while holding water, and they can always be washed with ethanol and remade into aerogels. Potato starch aerogels can also be pyrolyzed into carbon aerogels for higher-temperature aerospace applications. In summary, the green synthesis route, the biodegradability and recyclability of the material, and the desired properties of the potato starch aerogels in our design make them potential sustainable materials for aerospace applications. For future work, the hydrophobicity of the potato starch aerogels will be improved by the modification of the surface. It is pertinent to reduce possible adverse behavior like mold growth caused by the hydrophilicity of potato starch aerogels.

## 4. Materials and Methods

### 4.1. Materials Used for Aerogel Formulations

GEFEN pure potato starch was used in this research. 99.7% acetic acid and 99.5% glycerol were obtained from Sigma-Aldrich.(St. Louis, MO, USA). Distilled water was prepared in the lab.

### 4.2. Method for Fabricating Aerogel Including Supercritical Drying

The formulations of the potato starch aerogels are listed in Table 1. The potato starch concentration was varied from 5 to 10 wt%. The weight ratio of potato starch to acetic acid ranged from 25 to 75 g/g. The weight ratio of glycerol to potato starch ranged from 0.5 to 1 g/g. For example, run 1 was prepared as follows: a mixture of 5 g potato starch, 5 g glycerol, 0.063 mL (0.07g) acetic acid, and 39.93 mL (39.93 g) H_2_O was heated by an oil bath until it boiled and formed a viscous sol. The viscous sol was poured into a cylinder mold. The sol was aged at RT for 1 day and then put in freezer at −20 °C for 1 day, and the gels were extracted into ethanol and washed with ethanol daily for five times. The resulting gel was dried by supercritical extraction with CO_2_.

### 4.3. Experimental Design and Analysis

Design Expert, version 13 from Stat-Ease, Inc. (Minneapolis, MN, USA), was used to conduct the experimental design and analysis. Three variables (potato starch concentration, (5–10 wt%), potato starch/acetic acid ratio (25–75 g/g), and glycerol/potato starch ratio (0.5–1 g/g) were used in the experimental design. A total of 18 aerogel formulations with four repeats with central composite design were prepared, as shown in Table 1. Multiple linear regression was used to analyze the collected data. A full quadratic equation of the variables, including all two-way interactions, was considered for each response, and terms deemed not significant were eliminated from the model.

### 4.4. Bulk Density, Shrinkage, and Porosity Determination

The bulk density ρ (g/cm^3^) of each aerogel sample is calculated by its mass and volume,

(1) $$\rho =\frac{m}{v_{c}},$$

where m is sample mass (g), and $v_{c}=\pi d^{2}h/4$ is the volume (cm^3^), where d and h are the sample’s diameter and length, respectively.

The sample linear shrinkage $S\;\left(\%\right)$ is determined by the relative change in diameter,

(2) $$S\;\left(\%\right)=100\times \frac{d_{0}-d_{x}}{d_{0}},$$

where $d_{0}$ is the initial gel diameter and also the mold diameter. $d_{x}$ is the diameter of aerogel monoliths.

A Micromeritics Accupyc li 1345 (Micromeritics Instrument Corporation, Norcross, GA, USA) helium pycnometer was utilized to measure the skeleton density (ρ_s_) of the samples in a 10 cm^3^ micromeritics cell with a cap. The aerogel porosity $P_{t}\;(\%)$ was calculated as follows:

(3) $$P_{t}\;\left(\%\right)=100\times \left(1-\frac{\rho }{\rho_{s}}\right)\;$$

### 4.5. Instrument Characterization

Aerogel samples were degassed at 80 °C overnight for nitrogen adsorption–desorption testing using a Micromeritics Tristar 3020 II (Micromeritics Instrument Corporation, Norcross, GA, USA) in order to determine the BET surface areas, pore sizes, and absorption/desorption curves of the aerogel samples. Optical images of potato starch were obtained from KeyenceVHX Digital Microscope (Keyence, Itasca, IL, USA) with a 2000× magnification. Scanning electron microscopy (SEM) was used to obtain micrographs of the aerogels using a Hitachi S-4700 field emission microscope (Hitachi, Schaumburg, IL, USA) at 2 KV. Trident C-Therm (Fredericton, NB, Canada), conforming to ASTM D7984 [30] was used to conduct the measurement of the thermal conductivities of the aerogels. Thermogravimetric analysis (TGA) and derivative thermogravimetric analysis were performed with TA Instruments Q500 TGA (TA Instruments, New Castle, DE, USA) with a heat ramp rate of 10 °C/min and temperature heated up to 800 °C. Compressive testing was performed using an Instron electromechanical load frame (model 5584, Instron, Norwood, MA, USA) with Bluehill control software version 4.25. An Interface load cell with a full calibrated range of 500 lbf with a corresponding error of 1% of reading, or better, was used. The aerogel sample was compressed at a constant displacement rate of 0.05 in./min until 450 lbf was measured. Force and displacement data were collected at a frequency of 500 Hz during the compression. Young’s moduli were calculated from the stress–strain curve collected. Stress and strain were calculated using the below equations, where F is the force with unit lbf, ∆L is the length change during the test, and L is the original length of the aerogel.

(4) $$Stress=\frac{4.448\times 4\times F}{\pi {dx}^{2}}$$

(5) $$Strain\;(\%)=\frac{∆L}{L}$$

## Figures and Tables

**Table 1:** Formulation and properties of the potato starch aerogels.

Run	Potato Starch, wt%	Potato Starch/Acetic Acid	Glycerol/Potato Starch	Density, g/cm^3^	Shrinkage, %	Porosity, %	Surface Area, m^2^/g	Young’s Modulus, MPa	Thermal Conductivity, mW/mK
1	10	75	1	0.235	26.8	84.4	172.0	12.4	42.6
2	10	50	0.75	0.232	26.7	84.5	151.6	37.4	44.3
3	7.5	50	0.5	0.277	36.0	77.6	70.5	13.7	38.1
4	7.5	75	0.75	0.216	32.2	86.0	78.3	23.9	42.5
5	10	75	0.5	0.278	33.2	78.4	109.7	37.8	45.2
6	7.5	50	1	0.170	25.9	88.3	157.3	9.9	41.7
7	7.5	50	0.75	0.178	28.2	86.0	109.9	15.8	41.4
8	7.5	50	0.75	0.174	25.4	87.9	137.6	8.1	39.9
9	5	50	0.75	0.106	25.3	91.1	160.1	5.6	37.2
10	5	25	1	0.104	26.4	92.8	170.7	2.7	37.7
11	10	25	1	0.222	24.7	84.6	163.3	30.4	43.1
12	7.5	50	0.75	0.163	25.3	89.7	112.4	25.1	38.1
13	5	75	1	0.118	28.1	89.5	198.6	14.5	37.5
14	5	25	0.5	0.123	28.6	91.5	159.1	5.4	37.9
15	7.5	50	0.75	0.200	27.6	87.3	73.4	21.2	39.2
16	10	25	0.5	0.295	32.7	80.5	175.3	19.3	42.9
17	5	75	0.5	0.114	27.0	92.2	175.3	7.8	37.5
18	7.5	25	0.75	0.164	24.6	89.5	138.1	24.2	40.6

## Data Availability

The original contributions presented in this study are included in the article. Further inquiries can be directed to the corresponding author.
